# Ear disease, hearing loss, and cognitive outcomes in high school children who were previous participants in a randomized placebo controlled trial of an 11 valent conjugate pneumococcal vaccine administered in infancy

**DOI:** 10.1016/j.lanwpc.2024.101128

**Published:** 2024-07-31

**Authors:** Andrea S. Miele, Elisabeth D. Root, Phyllis Carosone-Link, Veronica Tallo, Marilla Lucero, Diozele Hazel Sanvictores, Yun Ye, Kenny H. Chan, Eric A.F. Simões

**Affiliations:** a Department of Pediatrics, University of Colorado School of Medicine, Aurora, CO, USA; b Institute for Disease Modeling, Bill & Melinda Gates Foundation, Seattle, WA, USA; c Department of Clinical Trials, Epidemiology and Biostatistics, Research Institute for Tropical Medicine Department of Health, Muntinlupa, Manila, Philippines; d Division of Epidemiology, College of Public Health, The Ohio State University, Columbus, OH, USA; e Department of Otolaryngology-Head and Neck Surgery, University of Colorado School of Medicine, Aurora, CO, USA; f Department of Pediatric Otolaryngology, Children's Hospital Colorado, Aurora, CO, USA; g Department of Epidemiology Center for Global Health, Colorado School of Public Health, Aurora, CO, USA

**Keywords:** Philippines, Adolescent health, School success, Epidemiology, Neuropsychology

## Abstract

**Background:**

The WHO estimates that close to 1.7 billion people worldwide have hearing loss; 34 million of whom are children, with 90% residing in low- and middle-income countries. While the effects of ear disease and hearing loss on language, academic, and behavioral development are established, there is remarkably little data on intellectual and other cognitive differences.

**Methods:**

Here we report results from an extension of a randomized controlled vaccination trial originally carried out between 2000 and 2004. Primary caregivers completed demographic and household questionnaires. Beginning in 2016, children were followed up for a hearing and ear disease evaluation. Participants also completed extensive cognitive testing, which included the domains of IQ, language, attention and processing speed, visual and visuospatial skills, and learning and memory. The association between ear disease and hearing loss and each of the cognitive outcomes was examined using multivariate linear regression models.

**Findings:**

We followed up 8926 children ages 14–19 years old. Children with hearing loss or ear disease had lower socioeconomic status compared to children without. However, even after controlling for a high number of covariates, all levels of ear disease or hearing loss were associated with clinically relevant reductions across all cognitive domains, though effect sizes were small. Even mild ear disease or hearing loss was associated with a −0.15 (95% CI: −0.20, −0.11) and a −0.23 (95% CI: −0.32, −0.14) standard deviation reduction, respectively, in IQ. The effects of ear disease and hearing loss were additive as children with both had the lowest cognitive scores.

**Interpretation:**

Untreated ear disease and hearing loss exert measurable effects on cognition that are able to be detected into the teenage years. Early identification of hearing loss and chronic ear disease may have lifelong benefits. Individuals with ear disease and/or hearing loss may require supports and services in addition to those related to speech and language therapy.

**Funding:**

The 10.13039/100000865Bill and Melinda Gates Foundation OPP1142570.

**Research in context**

**Evidence before this study**

The global burden of ear disease and hearing loss is high, and a significant proportion of this burden is among children in low- and middle-income countries. Cognitive effects associated with ear disease and hearing loss beyond language are surprisingly understudied in this vulnerable group; available evidence provides conflicting results. Cohort studies have found that otitis media is both related and unrelated to cognition in infancy/toddlerhood, young childhood, and in middle school children. Hearing loss is known to be positively associated with language, academic, and behavioral difficulties in early childhood. Some of this research, including a meta-analysis, finds reduced IQ in children with hearing loss, but still other studies report no differences. Other research focuses only on aspects of IQ such a verbal skills, but does not look further. Large cohort studies from western countries are available; however, these utilize behavioral questionnaires often completed by parents or teachers as proxies of functioning. Only a single study was able to be located investigating the relationship between hearing loss and cognitive difficulties in a low- and middle-income country (Serbia). As such, limitations of this existing literature include small sample sizes, truncated age ranges (i.e., only younger children included), and a focus on high income country populations.

**Added value of this study**

In this study, we present strong evidence of negative effects of ear disease and/or hearing loss on all measured cognitive domains after controlling for a significant number of potential confounds. Our study includes comprehensive cognitive testing results including IQ, language and verbal skills, attention and processing speed, visual and visuospatial skills, and both verbal and visual learning and memory in over 8000 teenagers in Bohol, Philippines. Results reveal decreases in nearly all individual subtests for participants with either ear disease or hearing loss, even in nonverbal and non-auditory skills. The biggest effects were seen for those with more severe hearing loss, though effects were additive. Ear and hearing health are thus important for the thinking skills beyond language that are also acquired and develop throughout childhood. Children with hearing loss or ear disease had lower socioeconomic status (as measured by a variety of wealth, education, and employment factors) compared to children with no hearing loss or ear disease.

**Implications of all the available evidence**

Ear disease and hearing loss are strongly related to overall cognitive development and are thus involved in children realizing their academic, economic, and social potential. Interventions for ear disease and hearing loss should include services beyond speech and language therapy; even older children would still benefit from supports. Children in low- and middle-income countries are at increased risk of this potentially preventable burden given the impact of even mild ear disease and hearing impairment on neurocognitive outcomes. Prevention of ear disease and hearing loss in early childhood could have major economic impacts in adulthood.

## Introduction

The WHO estimates that close to 1.7 billion people worldwide have hearing loss, 34 million of whom are children,^1^ with over 90% in low- and middle-income countries (LMIC), mainly Southeast Asia, the Western Pacific, and Africa.^2^ Chronic middle ear diseases such as suppurative otitis media are the main cause of mild to moderate hearing impairment in children. Otitis media causes fluctuating conductive hearing loss and is a well-known risk factor for early auditory development.^3^ Several large cohort studies have utilized parent and teacher report to underscore a relationship between otitis media and later academic and behavioral difficulties.^4^, ^5^, ^6^ A few other studies have examined the relationship between otitis media and cognition in children through intellectual ability (IQ) and/or auditory/verbal working memory. Results in the younger cohorts have been mixed: while older studies report limited association,^7^^,^^8^ more recent studies found positive associations.^9^, ^10^, ^11^, ^12^, ^13^ Specifically, increased time spent with otitis media or the more severe intervention required for otitis media treatment has been related to later decreased intellectual ability (IQ)^9^, ^10^, ^11^, ^12^, ^13^; however, only one small study found lasting effects of decreased IQ in teenage years.^11^ Potentially secondary to speech and language deficits, reduced verbal working memory has also been found in children with otitis media.^14^ Language deficits in children with hearing loss are well established in younger children,^15^ but broader thinking skills in older children, and especially children in LMIC, are rarely investigated.

Despite this controversial literature, there are remarkably few studies examining the effects of untreated ear disease and/or hearing loss on overall cognitive development in older children, and only a single study in children from a LMIC.^16^ A follow up to a larger randomized controlled vaccination trial, in which we collected hearing, ear disease, and cognitive data on a very large cohort of older children in the Philippines, allowed us to address this lacuna. We hypothesized that children with greater ear disease and/or hearing loss will display more deficits in cognitive functioning, particularly in the areas of overall intellectual functioning, auditory attention/working memory, language and verbal reasoning, and verbal learning and memory.

## Methods

### Study design

This is a 14–18-year follow-up of children enrolled in a randomized placebo-controlled trial of an 11-valent pneumococcal conjugate vaccine conducted between 2000 and 2004 in Bohol, Philippines (ARIVAC Study^17^). This study was reviewed and approved by the Colorado Multiple Institutional Review Board (protocol #16-0473) and by the Research Institute for Tropical Medicine Institutional Review Board (protocol #2016-20).

### Study population

The original ARIVAC cohort was located through intensive in-person follow-up and migrant tracking. Briefly, the home address of each child enrolled in the original ARIVAC trial was visited by field personnel. If the child still resided at the address, they were asked to participate in the study. If the child was no longer at the address, neighbors were asked where the family had moved and for contact information if available. Field personnel made several attempts to contact the family, either by visiting the last known address or by calling mobile phones.

### Study procedures

Once an ARIVAC child and family had been located, informed consent from parents, and assent from children under the age of 18 or consent for children 18 or older, was obtained before data collection. After consenting, one parent or primary caregiver was asked to complete a questionnaire that asked about general demographics, parental occupation and employment, household size and composition, environmental risk factors (such as smoking, crowding, cooking fuel, and housing type), and the child's medical history. Children were asked to come to the study office in Tagbilaran for otoscopic screening and to check for persistent cleft palate, repaired cleft palate, and sub mucus cleft as potential confounders. Children without the above proceeded to ear cleaning, after which tympanometry and distortion product otoacoustic emissions (DPOAEs) were completed by trained nurses followed by audiologic screening and evaluation.^18^ Children also completed a cognitive assessment consisting of measures of verbal reasoning and language processing, visual and visuospatial reasoning, attention/working memory, processing speed, and verbal and visual learning and memory.

### Exclusion criteria

Children born with congenital malformation were excluded from this study. Children reported to have chronic neurological conditions (i.e., epilepsy, hydrocephalus, paralysis) were also excluded.

### Outcomes

The primary study outcomes consisted of 14 selected subtests modeled after those used routinely in pediatric cognitive evaluation^19^, ^20^, ^21^ to assess overall intellectual ability, verbal skills and language comprehension, visual/visuospatial reasoning, auditory attention/working memory, processing speed, and verbal and visual learning and memory. All tests were translated into Visayan, the primary language spoken in Bohol, Philippines. Included items underwent two rounds of pilot-testing in the community for validation. Several measures contained items that were removed due to inability for the item to be successfully translated. Cognitive testers underwent extensive training, including GCP compliance, team review of instructions, and practice role play before administering the measures to study participants. Certain tests were then combined into overarching indices (i.e., overall IQ) per existing standardized testing protocols for analysis. From the raw scores, we constructed age-group-specific z-scores for each cognitive outcome and standardized using the placebo group population means. Prior analysis found that the vaccine reduced both hearing and ear disease. In order to avoid introducing bias, we used the placebo group means because this group would not have had the benefits of the vaccine. The z-scores were calculated as: (individual score—placebo population mean score)/placebo population standard deviation), where the mean and SD were calculated within each age group. All results are shown in standard deviation units.

### Covariates

#### Hearing

Hearing thresholds were obtained using results of the portable audiometer (HearScreen or HearTest) or the Bohol Hearing Center (referred for failed hearing screening).^22^ Three-frequency (1K, 2k, and 4K) pure tone average (PTA) for air conduction (AC) was computed. Five categories of hearing loss severity were cataloged as previously described.^23^ Because only 663 subjects were identified to have hearing loss, AC thresholds of the cohort were collapsed into three categories to aid in analyses: 1) no hearing loss, 2) mild hearing loss (mild), and 3) moderate/severe/profound hearing loss (m/s/p). Bone conduction was not included.

#### Ear disease

Ear disease was classified using a flowchart-based algorithm^22^ that included results of an ear exam (e.g., ear pain, otorrhea), tympanogram (e.g., ear canal volume), and review of video otoscopy by ENTs and their indication of 15 ear signs and symptoms. Ear disease severity was classified into four categories: no ear disease, mild ear disease (acute otitis media, otitis media with effusion, healed perforation of the tympanic membrane, or myringosclerosis), moderate ear disease (dry perforation of the tympanic membrane or adhesive otitis media), or severe ear disease (chronic suppurative otitis media). For the current analysis, participants were grouped into three categories: 1) no ear disease, 2) mild ear disease (mild), and 3) moderate/severe ear disease (m/s) in the worse ear, correlating to a classification schema proposed by Mammarella et al.^24^

#### Parental questionnaire

On enrollment, a parent or primary caregiver was verbally administered a questionnaire by trained field staff. Socioeconomic and behavioral characteristics of children with hearing loss/otitis media have been reported to explain a significant portion of variance in overall thinking skills and developmental outcomes.^25^^,^^26^ Therefore, a number of potential confounding factors were considered: child's birthweight, whether the child had a chronic health condition (e.g., asthma, heart condition, respiratory disease, gastrointestinal, kidney and blood conditions, etc.), total number of people in the child's household, total number of adults (>15 years old), and total number of children (≤15 years old) in the household, household crowding (more than 2 people per room), total number of children ever born to the mother, child birth order (first, second to fourth, fifth or more), whether the child attended preschool, parent's marital status (married, separated/divorced, never married), highest education attained by either parent (elementary school or less, high school or vocational school, some college, college or post graduate), parental employment (manager/professional/technical, clerical/service, agricultural/crafts/skilled manual labor, unskilled labor/farmer, missing), whether the mother had health insurance, urban residence and a household wealth index (developed using the DHS methodology).^27^

### Statistical analysis

Data from all participants who completed the parental questionnaire, returned for the ear exam and had a valid result, and completed at least one cognitive test module, were collected and managed using a REDCap^28^ database, stored at the University of Colorado Denver, and included in the analysis. Completion rates were very high for the parental questionnaire, so we adopted an available case approach to missing data after ensuring that covariates did not differ between included and excluded cases. To assess the relationship between the mean score for each cognitive outcome and the different levels of ear disease and hearing loss, we calculated p-values using ANOVA. The relationship between child and family characteristics and different levels of ear disease and hearing loss was assessed using ANOVA (continuous variables) or chi-squared tests (categorical variables). To assess which covariates from the parental questionnaire were associated with cognitive outcomes, hearing loss, and ear disease, we conducted extensive univariate regression analysis and covariates with a p < 0.1 were initially considered for multivariate models (Table S1). The association between hearing loss and ear disease and each of the cognitive outcomes was examined using multivariate linear regression models. We conducted an extensive model selection process that included entering all covariates into our model and sequentially removing those with no significant effect. Given the large number of covariates, we assessed variable multicollinearity using VIF and correlation coefficients. Several additional robustness checks were conducted. Separate multivariate models for hearing loss and ear disease and one with an interaction effect between ear disease and hearing loss were examined. The final model presented here was the best fit (as assessed by adjusted R2 and RMSE) and included the main effects of hearing loss and ear disease, but not the interaction effect (which was not statistically significant in any model). All statistical analyses were performed using R v4.1.1.^29^

### Role of the funding source

This study was supported by the Bill and Melinda Gates Foundation (grant number: OPP1142570) and Colorado CTSA (grant numbers: UL1TR002535). The funders did not participate in any aspect of the study, including study conduct, data collection, analyses of the data, or the writeup of the manuscript. All authors confirm that they had full access to all the data in the study and had final responsibility for the decision to submit for publication.

## Results

**Fig. 1:**
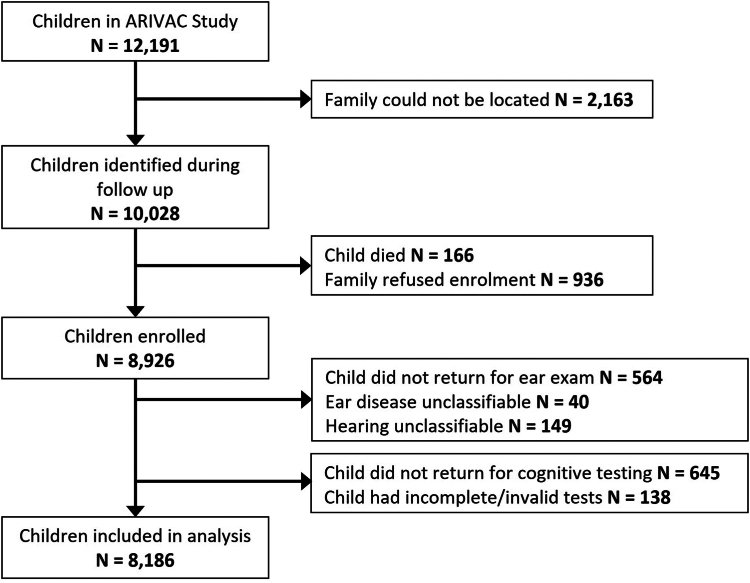
Follow-up of study subjects by Intervention arm, from the original ARIVAC study to the current study of 11PCV.

Participants were studied between September 1, 2016 and February 1, 2020. Of the 12,191 eligible ARIVAC trial participants, 8926 (73.2%) were enrolled in the follow-up study and completed the parental questionnaire (Fig. 1). Of the 8926 children enrolled in the study, 8362 returned for the ear exam. Some additional loss to follow-up occurred due to unclassifiable hearing (e.g., if participants were unable to follow instructions for the audiometry portion) or ear exams, leading to a final sample of 8321 children with both a parental questionnaire and a valid ear/hearing exam. Eight thousand two hundred thirteen children that completed the ear exam also completed at least one module in the cognitive assessment. Once covariates were considered, 8186 children were included in the analysis, though not all of them completed or had valid results for all 14 cognitive tests.

**Fig. 2:**
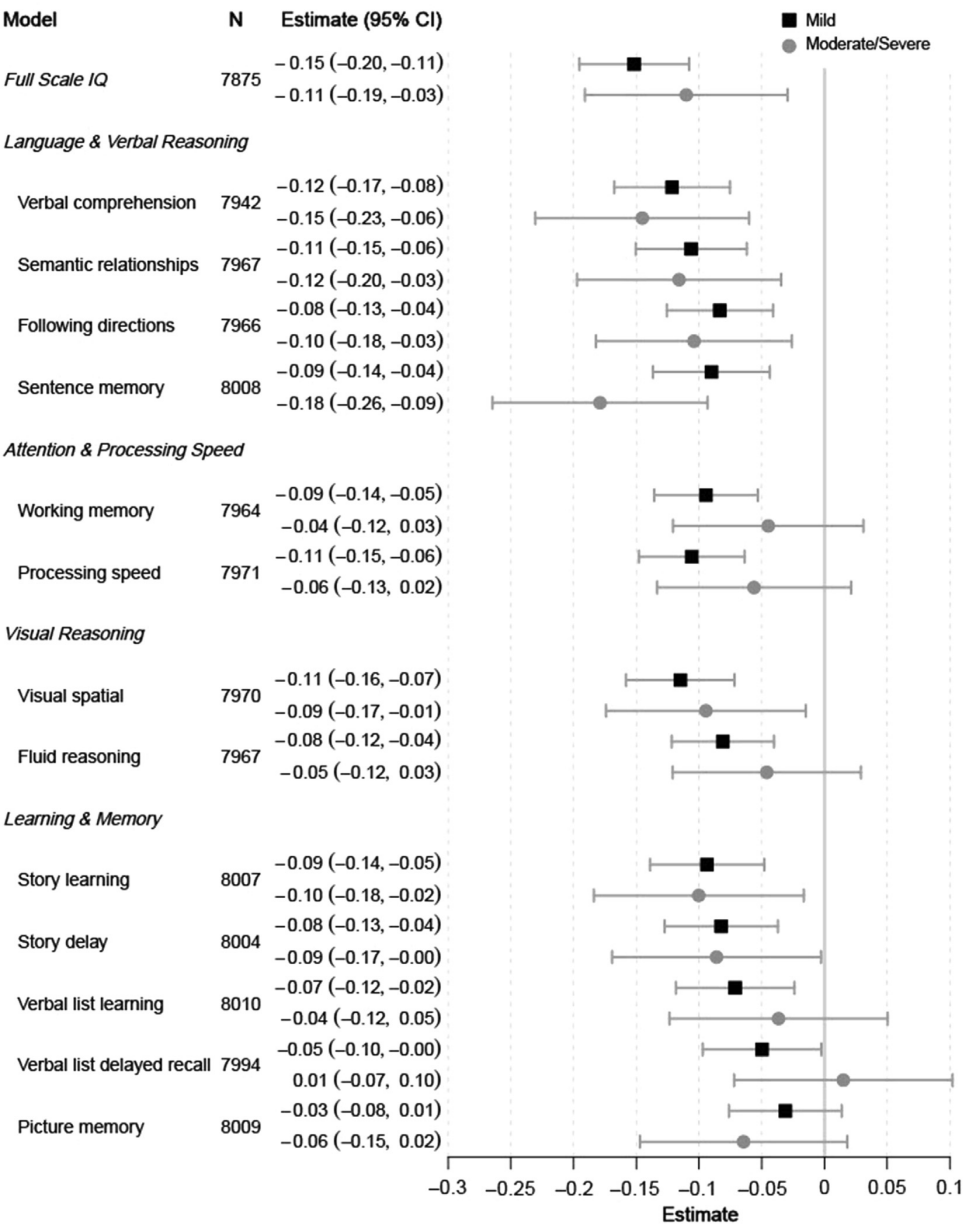
Estimates (in standard deviation units) and 95% confidence intervals for the effect of mild ear disease (black square) and moderate/severe ear disease (grey circle) on cognition by domain. Adjusted models include additional covariates hearing loss, household SES, crowding, total births to the mother, preschool attendance, child birth order, urban residence, parental marital status, education and employment. Note: Ear disease severity was classified accordingly: no ear disease, mild ear disease (acute otitis media, otitis media with effusion, healed perforation of the tympanic membrane, or myringosclerosis), moderate ear disease (dry perforation of the tympanic membrane or adhesive otitis media), or severe ear disease (chronic suppurative otitis media). Note: Hearing Loss was classified accordingly: no hearing loss, mild hearing loss (16–30 dB PTA), moderate hearing loss (31–60 dB PTA), severe hearing loss (61–80 dB PTA), or profound hearing loss (>80 dB PTA).

**Fig. 3:**
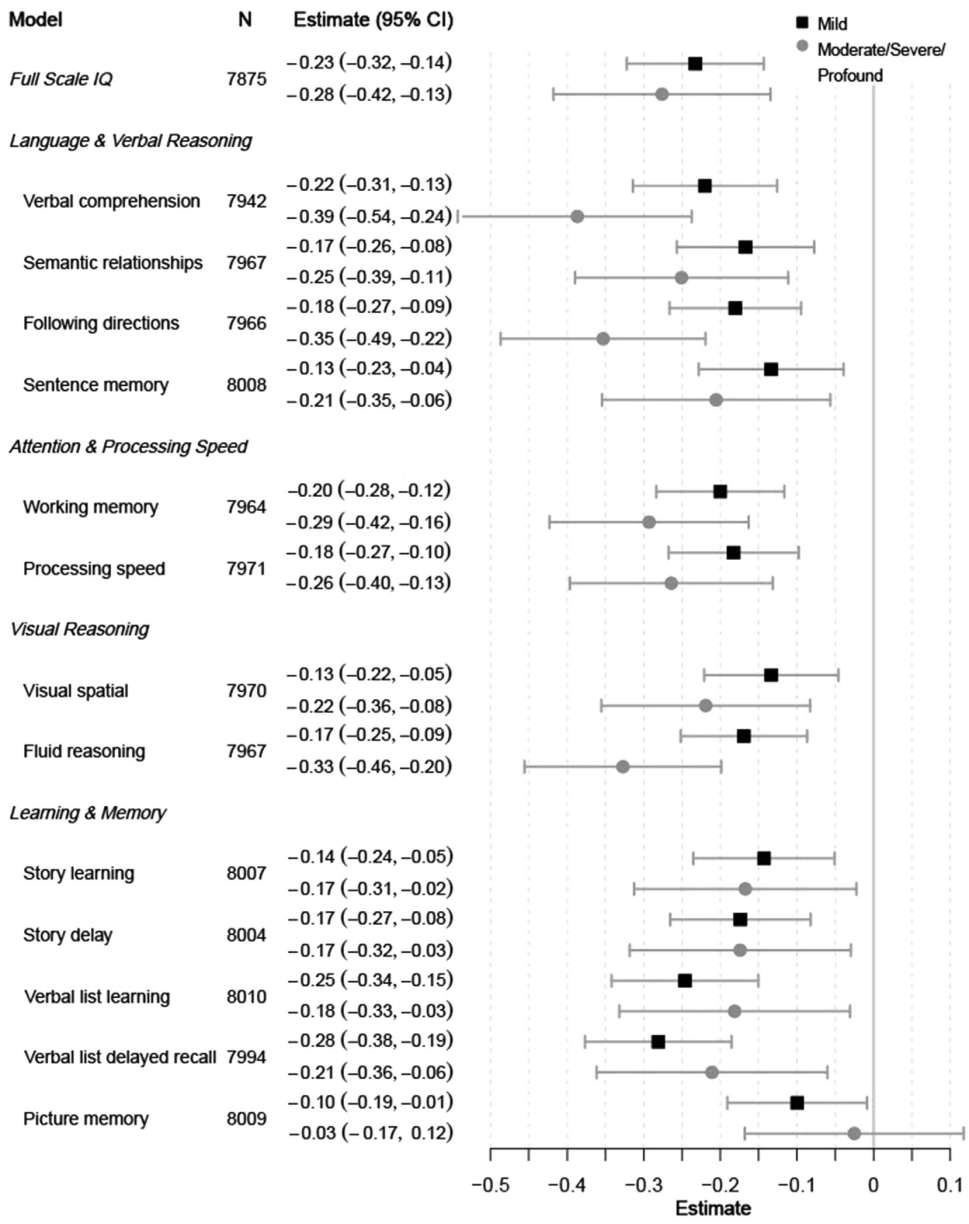
Estimates (in standard deviation units) and 95% confidence intervals for the effect of mild hearing loss (black square) and moderate/severe/profound hearing loss (grey circle) on cognition by domain. Adjusted models include additional covariates ear disease, household SES, crowding, total births to the mother, preschool attendance, child birth order, urban residence, parental marital status, education and employment. Note: Ear disease severity was classified accordingly: no ear disease, mild ear disease (acute otitis media, otitis media with effusion, healed perforation of the tympanic membrane, or myringosclerosis), moderate ear disease (dry perforation of the tympanic membrane or adhesive otitis media), or severe ear disease (chronic suppurative otitis media). Note: Hearing Loss was classified accordingly: no hearing loss, mild hearing loss (16–30 dB PTA), moderate hearing loss (31–60 dB PTA), severe hearing loss (61–80 dB PTA), or profound hearing loss (>80 dB PTA).

Descriptive features of the sample by ear disease and hearing loss classification are presented in Table 1. Simple univariate analysis shows significant differences in scores across all cognitive domains when comparing children with no ear disease, mild ear disease, and moderate/severe ear disease as well as between children with no hearing loss, mild hearing loss, and moderate/severe/profound hearing loss. There were also significant differences between groups across many of the study covariates including attending preschool, parental education, and parental employment. Univariate associations between each study covariate and each cognitive outcome are shown in Table S1 in the Supplemental Appendix. Table 2 and Figs. 2 and 3 show the point estimates and 95% CIs for the effects of hearing loss and ear disease from multivariate models of each cognitive domain.

**Table 1:** Descriptive statistics of the study sample.

	Total sample N = 8097			No ear disease N = 4537			Mild ear disease N = 2937			Moderate/Severe ear disease N = 623			p^a^	No hearing loss N = 7448			Mild hearing loss N = 465			Moderate/Severe/Profound hearing loss N = 184			p^a^
	N	Mean	SD	N	Mean	SD	N	Mean	SD	N	Mean	SD		N	Mean	SD	N	Mean	SD	N	Mean	SD	
**Cognitive outcomes**																							
Full scale IQ	7932	191.7	33.1	4446	195.4	32.5	2882	187.3	32.5	604	186	36.9	**<0.001**	7315	192.7	32.8	448	180.9	35.2	169	180.3	35.8	**<0.001**
Language and verbal reasoning																							
*Verbal comprehension*	7999	41.6	8.8	4491	42.4	8.4	2901	40.7	9	607	39.7	9.9	**<0.001**	7377	41.8	8.6	451	39.3	9.6	171	38.1	10.8	**<0.001**
*Semantic relationships*	8031	4.1	4.5	4493	4.5	4.6	2918	3.7	4.2	620	3.4	4.1	**<0.001**	7387	4	4.5	462	3.0	3.9	182	2.7	3.6	**<0.001**
*Following directions*	8031	17.5	4.5	4493	17.9	4.4	2918	17.1	4.5	620	16.6	5	**<0.001**	7387	18	4.4	462	16.4	5.2	182	15.6	5.5	**<0.001**
*Sentence memory*	8067	16.5	3.3	4522	16.7	3.2	2925	16.3	3.3	620	15.8	3.7	**<0.001**	7428	17	3.2	462	15.9	3.7	177	15.7	4.2	**<0.001**
Attention and processing speed																							
*Working memory*	8031	25.4	5.6	4493	25.8	5.4	2918	24.9	5.6	620	24.7	6.1	**<0.001**	7387	26	5.5	462	23.8	6.2	182	23.4	7.3	**<0.001**
*Processing speed*	8030	61.4	16.4	4492	62.8	16.3	2918	59.7	16.1	620	59.5	17.3	**<0.001**	7386	62	16.2	462	57.0	17.1	182	55.7	19.2	**<0.001**
Visual reasoning																							
*Visual spatial*	8031	29.6	8.8	4493	30.3	8.8	2918	28.8	8.5	620	28.5	9.4	**<0.001**	7387	29.8	8.6	462	27.7	9.7	182	26.9	9.9	**<0.001**
*Fluid reasoning*	8031	32.9	7.6	4493	33.5	7.6	2918	32.2	7.5	620	31.9	7.8	**<0.001**	7387	33	7.5	462	30.8	7.8	182	29.7	8.9	**<0.001**
Learning and memory																							
*Story learning*	8066	24.7	9.9	4522	25.4	9.9	2924	23.8	9.7	620	23.2	10.1	**<0.001**	7427	25	9.9	462	22.6	10.2	177	22.6	9.8	**<0.001**
*Story delay*	8063	20.6	10.2	4520	21.3	10.3	2923	19.8	10	620	19.1	10.4	**<0.001**	7424	21	10.2	462	18.1	10.3	177	18.3	10	**<0.001**
*Verbal list learning*	8069	35.5	7.7	4523	35.9	7.6	2926	35.1	7.8	620	34.9	8.3	**<0.001**	7428	36	7.6	463	33.6	8.6	178	34.2	9	**<0.001**
*Verbal list delayed recall*	8052	9.6	3.1	4514	9.7	3.0	2920	9.5	3.1	618	9.5	3.3	**0.004**	7411	10	3	463	8.8	3.3	178	9.0	3.6	**<0.001**
*Picture memory*	8068	22.6	6.7	4522	22.8	6.7	2925	22.4	6.7	621	22	6.9	**0.003**	7426	23	6.7	463	21.4	6.9	179	21.9	7.6	**<0.001**
**Family/Child characteristics**																							
Child's birthweight	8075	2925.3	597.8	4528	2919.7	587.7	2925	2928.7	616.1	622	2950.3	583.9	0.543	7427	2925.7	596.5	464	2924.4	619.9	184	2911.2	598.8	0.621
Total births of mother	8095	4.4	2.3	4536	4.2	2.1	2936	4.7	2.4	623	5	2.5	**<0.001**	7446	4.4	2.3	465	4.7	2.3	184	4.7	2.5	**0.008**
Number of children <15 years in the household	8089	1.6	1.5	4534	1.5	1.4	2932	1.7	1.6	623	1.7	1.5	**<0.001**	7440	1.6	1.5	465	1.6	1.5	184	1.6	1.4	0.774
Number of people in the household	8096	6.5	2.6	4537	6.2	2.4	2936	6.7	2.7	623	6.8	2.7	**<0.001**	7447	6.4	2.6	465	6.6	2.5	184	6.5	2.4	0.891
Household SES index	8097	0.01	1	4537	0.1	1	2937	−0.1	0.9	623	−0.1	0.9	**<0.001**	7448	0.03	1	465	−0.2	0.8	184	−0.2	0.9	**<0.001**
Crowding	8074	2.8	1.7	4525	2.6	1.6	2928	3	1.8	621	3	1.8	**<0.001**	7426	2.8	1.7	464	3	1.8	184	2.9	1.7	**0.016**
	**N**	**%**		**N**	**%**		**N**	**%**		**N**	**%**		**p**	**N**	**%**		**N**	**%**		**N**	**%**		**p**
Child has a chronic health condition	240	3		132	2.9		87	3		21	3.4		0.346	219	2.9		7	1.5		14	7.6		**<0.001**
Child attended preschool	3219	39.8		1948	43		1062	36.2		209	33.7		**<0.001**	3015	40.5		138	29.7		66	36		**<0.001**
Mother has health insurance	5779	71.4		3307	72.9		2062	70.2		410	65.8		**0.002**	2017	27.1		140	30.1		58	31.5		0.697
Child's birth order																							
*First*	2209	27.3		1414	31.2		668	22.8		127	20.4		**<0.001**	2063	27.7		97	20.9		49	26.6		**0.011**
*Second to fourth*	4399	54.3		2396	52.8		1651	56.2		352	56.5			4038	54.2		265	57.0		96	52.2		
*Fifth or more*	1487	18.4		726	16		617	21		144	23.1			1345	18.1		103	22.2		39	21.2		
Parent's marital status																							
*Never married*	306	3.8		177	3.9		105	3.6		24	3.9		**0.028**	281	3.8		17	3.7		8	4.3		0.294
*Separated/divorced*	1036	12.8		539	11.9		397	13.5		100	16.1			935	12.6		77	16.6		24	13.0		
*Married*	6734	83.4		3806	84.2		2429	82.9		499	80.1			6211	83.6		371	79.8		153	82.6		
Parental education (highest)																							
*College or post grad*	2575	31.8		1595	35.2		823	28.1		157	25.2		**<0.001**	2439	32.8		92	19.8		44	23.9		**<0.001**
*Some college*	1338	16.5		719	15.9		512	17.5		107	17.2			1213	16.3		95	20.4		30	16.3		
*HS or vocational school grad*	2138	26.4		1170	25.8		797	27.2		171	27.5			1948	26.2		140	30.1		50	27.2		
*Elementary school or less*	2034	25.2		1051	23.2		796	27.2		187	30.1			1836	24.7		138	29.7		60	32.6		
Parental employment (highest)																							
*Manager/Professional/Technical*	1269	15.7		799	17.6		388	13.2		82	13.2		**<0.001**	1212	16.3		35	7.5		22	12.0		**<0.001**
*Clerical/Service/Armed Forces*	3427	42.3		1932	42.6		1264	43		231	37.1			3148	42.3		204	43.9		75	40.8		
*Agriculture/Crafts/Skilled labor/Plant or machine operator or assembler*	1514	18.7		827	18.2		559	19		128	20.5			1380	18.5		99	21.3		35	19.0		
*Unskilled labor/Farmer*	1237	15.3		632	13.9		483	16.4		122	19.6			1113	14.9		89	19.1		35	19.0		
Mother or father is deceased	623	7.7		325	7.2		249	8.5		49	7.9		0.233	563	7.6		48	10.3		12	6.5		0.125
Child lives with																							
*One parent*	1412	17.4		778	17.2		525	17.9		109	17.5		0.459	1294	17.4		86	18.5		32	17.4		0.4761
*Both parents*	6138	75.8		3459	76.3		2217	75.5		462	74.2			5659	76		339	72.9		140	76.1		
*Foster, grandparent, aunt/uncle, sibling, other*	543	6.7		298	6.6		193	6.6		52	8.3			491	6.6		40	8.6		12	6.5		
Urban residence	3284	40.6		1782	39.3		1213	41.3		289	46.4		**0.002**	3071	41.2		115	33.3		58	31.5		**<0.001**

Note: Ear disease severity was classified accordingly: no ear disease, mild ear disease (acute otitis media, otitis media with effusion, healed perforation of the tympanic membrane, or myringosclerosis), moderate ear disease (dry perforation of the tympanic membrane or adhesive otitis media), or severe ear disease (chronic suppurative otitis media). Bold values indiacte p < 0.05.

Note: Hearing Loss was classified accordingly: no hearing loss, mild hearing loss (16–30 dB PTA), moderate hearing loss (31–60 dB PTA), severe hearing loss (61–80 dB PTA), or profound hearing loss (>80 dB PTA).

Sample size, mean and standard deviation for cognitive outcomes and sample size and percent of sample for family and child characteristics by the total sample, by ear disease classification (none, mild, moderate/severe), and hearing loss classification (none, mild, moderate/severe/profound).

a p-value is the difference between groups (e.g., no, mild, moderate/severe) from ANOVA (continuous variables) or chi-squared tests (categorical variables).

**Table 2:** Estimated coefficients and 95% confidence intervals the effect of ear disease and hearing loss on cognitive development, from adjusted regression models by cognitive domain.

	N	Mild ear disease			Moderate/Severe ear disease			Mild hearing loss			Moderate/Severe/Profound hearing loss		
		Est	95% CI	p-value	Est	95% CI	p-value	Est	95% CI	p-value	Est	95% CI	p-value
**Full scale IQ**	7875	−0.152	(−0.196, −0.108)	**<0.001**	−0.110	(−0.191, −0.029)	**0.008**	−0.233	(−0.322, −0.143)	**<0.001**	−0.276	(−0.418, −0.134)	**<0.001**
**Language and verbal reasoning**													
Verbal comprehension	7942	−0.121	(−0.168, −0.075)	**<0.001**	−0.145	(−0.231, −0.060)	**0.001**	−0.220	(−0.314, −0.126)	**<0.001**	−0.387	(−0.536, −0.238)	**<0.001**
Semantic relationships	7967	−0.106	(−0.151, −0.062)	**<0.001**	−0.116	(−0.197, −0.035)	**0.006**	−0.167	(−0.257, −0.078)	**<0.001**	−0.251	(−0.390, −0.111)	**0.001**
Following directions	7966	−0.083	(−0.126, −0.041)	**<0.001**	−0.104	(−0.182, −0.026)	**0.010**	−0.180	(−0.266, −0.094)	**<0.001**	−0.353	(−0.487, −0.219)	**<0.001**
Sentence memory	8008	−0.090	(−0.137, −0.043)	**<0.001**	−0.179	(−0.265, −0.093)	**<0.001**	−0.134	(−0.228, −0.039)	**0.006**	−0.205	(−0.354, −0.057)	**0.007**
**Attention and processing speed**													
Working memory	7964	−0.095	(−0.136, −0.053)	**<0.001**	−0.045	(−0.121, 0.031)	0.248	−0.200	(−0.284, −0.117)	**<0.001**	−0.293	(−0.423, −0.163)	**<0.001**
Processing speed	7971	−0.106	(−0.148, −0.064)	**<0.001**	−0.056	(−0.133, 0.021)	0.155	−0.183	(−0.268, −0.098)	**<0.001**	−0.264	(−0.396, −0.132)	**<0.001**
**Visual reasoning**													
Visual spatial	7970	−0.115	(−0.158, −0.072)	**<0.001**	−0.095	(−0.174, −0.015)	**0.020**	−0.134	(−0.221, −0.046)	**0.003**	−0.219	(−0.355, −0.083)	**0.002**
Fluid reasoning	7967	−0.081	(−0.122, −0.040)	**<0.001**	−0.046	(−0.121, 0.029)	0.230	−0.169	(−0.252, −0.087)	**<0.001**	−0.327	(−0.456, −0.199)	**<0.001**
**Learning and memory**													
Story learning	8007	−0.094	(−0.139, −0.048)	**<0.001**	−0.100	(−0.184, −0.017)	**0.019**	−0.143	(−0.235, −0.051)	**0.003**	−0.167	(−0.313, −0.022)	**0.024**
Story delay	8004	−0.082	(−0.127, −0.037)	**<0.001**	−0.086	(−0.169, −0.003)	**0.043**	−0.174	(−0.266, −0.082)	**<0.001**	−0.174	(−0.318, −0.030)	**0.019**
Verbal list learning	8010	−0.071	(−0.118, −0.024)	**0.004**	−0.037	(−0.124, 0.050)	0.410	−0.246	(−0.342, −0.150)	**<0.001**	−0.181	(−0.332, −0.031)	**0.019**
Verbal list delayed recall	7994	−0.050	(−0.097, −0.002)	**0.040**	0.015	(−0.072, 0.102)	0.735	−0.281	(−0.377, −0.185)	**<0.001**	−0.211	(−0.361, −0.060)	**0.007**
Picture memory	8009	−0.031	(−0.076, 0.014)	0.174	−0.064	(−0.147, 0.018)	0.127	−0.100	(−0.191, −0.009)	**0.032**	−0.025	(−0.168, 0.118)	0.730

Note: Models include both ear disease and hearing loss as covariates and are adjusted for household SES, crowding, total births to the mother, preschool attendance, child birth order, urban residence, parental marital status, education and employment. Bold values indiacte p < 0.05.

Note: Ear disease severity was classified accordingly: no ear disease, mild ear disease (acute otitis media, otitis media with effusion, healed perforation of the tympanic membrane, or myringosclerosis), moderate ear disease (dry perforation of the tympanic membrane or adhesive otitis media), or severe ear disease (chronic suppurative otitis media).

Note: Hearing Loss was classified accordingly: no hearing loss, mild hearing loss (16–30 dB PTA), moderate hearing loss (31–60 dB PTA), severe hearing loss (61–80 dB PTA), or profound hearing loss (>80 dB PTA).

Children with any degree of ear disease performed worse in every domain of cognitive functioning when compared to children with no ear disease; however, most effects were small regardless of level of ear disease. Specifically, overall IQ was reduced by 0.15 standard deviation for children with mild ear disease (95% CI: −0.20, −0.11) and found to be 0.11 standard deviation lower for children with moderate/severe ear disease (95% CI: −0.19, −0.03). In the language and verbal reasoning domains, participants with moderate/severe ear disease performed slightly worse on every measure than those with mild ear disease evidenced by slightly more negative effects; the sentence memory task was most related to disease as shown by the lowest scores obtained (−0.18; 95% CI: −0.26, −0.09). In the attention/processing speed and visual reasoning domains, individuals with mild ear disease actually performed worse than those with moderate/severe ear disease, though the confidence intervals between the two groups overlap suggesting a similar effect size across groups. In the learning and memory domain, performance was similarly reduced among both groups of ear disease and most impaired on a story learning and recall measure. On a list learning and recall measure, participants with mild ear disease again performed worse than their moderate/severe counterparts, though this may, again, reflect sample size as fewer individuals had moderate/severe disease. On a visual learning and memory task, level of ear disease was not associated with performance.

Effect sizes for hearing loss overall are generally small to medium, with the largest observed effect sizes emerging for the highest levels of hearing loss. Thus, compared to any level of ear disease, even individuals with mild hearing loss achieved lower scores. Specifically, children displayed a −0.23 standard deviation reduction in IQ with mild hearing loss and a −0.27 reduction in IQ with moderate/severe/profound hearing loss. In the language and verbal reasoning domain, performance on all four measures was negatively impacted, with individuals with worse hearing loss achieving the lowest scores (for example, verbal comprehension mild: −0.22; 95% CI: −0.314, −0.126; m/s/p: −0.387; 95% CI: −0.536, −0.238). On the two measures of attention and processing speed, individuals with worse hearing loss performed slightly worse; however, these reductions are especially interesting for the processing speed measure that does not have any verbal or auditory component with the exception of the instructions. Performance on measures of visual reasoning was also lower for any type of hearing loss, but slightly more so for individuals with moderate/severe/profound levels, which is again surprising as the only language involved is the directions. While effect sizes were smaller and more similar across hearing levels in the learning and memory domain, the biggest impact was observed in verbal list delayed recall (mild: −0.281; 95% CI: −0.377, −0.185; m/s/p: −0.211; 95% CI: −0.361, −0.060). Expected and less impact of hearing loss was observed on a measure of visual learning and recall (mild: −0.10; 95% CI: −0.19, −0.01; m/s/p: −0.03; 95% CI: −0.17, −0.12); however, this is again believed to reflect the small size of the highest level of hearing loss group. Results for all covariates in the full models are shown in Table S2.

## Discussion

The results of this study indicate a significant negative impact of all severities of ear disease and all levels of hearing loss in the four cognitive domains assessed, and on nearly every individual measure. Importantly, children with any hearing loss performed more poorly than children with ear disease in nearly all domains. Consistent with our hypotheses, hearing loss was most strongly related to language and verbal reasoning skills; the more severe the hearing loss, the greater the impairment in functioning. Ear disease was also associated with worse cognitive skills in most domains, though less impressively and consistently. Specifically, children were less able to define words, determine an overarching verbal concept, effectively interpret meaning of verbal/written instructions, and repeat back complex sentences. Interestingly, performance was equally low on “knowledge based” measures (i.e., expressive vocabulary, verbal concept formation) that develop throughout childhood. This evidence suggests influences of ear disease and hearing on the language aspects of intellectual development. Both ear disease and hearing loss also exerted influences on considered “active” auditory/verbal processes (i.e., receptive language comprehension, auditory processing/working memory), which means that these children are also less able to take verbal information in and hold it in mind, follow multistep commands, or comprehend more complex language.

Interestingly, children with hearing loss and ear disease also had slower overall processing speed, which is measured by the ability to complete a rote task as quickly as possible. The task requires no language or auditory processing and, as such, provides further evidence of the broad effects of ear disease and hearing loss on aspects of cognitive functioning. Further surprising, performance on the visual and visuospatial aspects of IQ were also negatively associated with ear disease and hearing loss, though to a lesser extent than the language and verbal reasoning domains. Taken together, ear disease and hearing loss therefore exert prominent effects on reasoning skills *overall*, and not solely on aspects of cognition thought to be obtained through exposure to auditory stimuli throughout development.

Lastly, children with ear disease and hearing loss also performed more poorly across measures of learning and memory, though some differences are noted. First, children with ear disease performed worse on story learning and memory vs. list learning and memory while those with hearing loss struggled on both of these measures. Thus, as hypothesized, children with hearing loss have a harder time effectively learning a list of disparate details, which relies on auditory working memory, as opposed to a story that is pre-organized (i.e., with a beginning, middle, and end), and where certain details prompt other details. Children with ear disease did not differ in their performance on a measure of visual learning and memory; however, children with mild hearing loss had a harder time.

One potential confounder to our results could be actual effects of hearing loss on the accuracy of the cognitive testing (i.e., Harbinger or overdiagnosis effect).^30^ However, any error introduced is believed to be small given the prior ear cleaning, testing room set-up, as well as factors embedded in the test administration itself. All children who completed testing had their ears cleaned first for the audiology portion. Then, testing was completed in a quiet room where the participant worked one-on-one with the examiner. In terms of the tests themselves, examiners were trained to not move forward with test items until understanding of the task itself had been achieved, and “training” items were built in to assure the examiner that the participant understood the task instructions. If difficulties in understanding still emerged, repetition, scaffolding, and/or demonstration of task instructions was permitted. Instances where a participant would need to respond to active feedback under time pressure to answer a question from an examiner do not occur. Robustness of this testing paradigm is reflected in essentially equivalent decreases in performance observed on the visual reasoning and processing speed measures that have a limited language component, including the instructions.

The negative effects of hearing loss on speech and language development are well established, with greater impacts on typical development observed the earlier age of onset of hearing difficulty. Children with hearing loss struggle to acquire vocabulary through both oral and written language.^31^ Even those with only unilateral hearing loss experience delays in acquisition of early reading skills,^32^ and those with only mild sensorineural hearing loss are also found to “lag” behind same age peers in academic achievement and to have failed at least one grade.^33^ Surprisingly, studies that have investigated intellectual deficits in children with hearing loss remain limited to reports about unilateral hearing loss. Specifically, a single meta-analysis consisting of four studies^34^ found children with unilateral hearing loss to have lower intellectual functioning. Interestingly, IQ differences were driven by performance on *nonverbal* measures as differences in verbal measures were more variable but still significant. Three of the four included studies^31^^,^^35^^,^^36^ were conducted in the United States, and only in young children (median age around 9-years). In addition to this meta-analysis, three additional studies were located, though they offer conflicting findings. While Dokovic et al. conducted their study in a middle-income country (Serbia) with a modest sample size of 144 children, they only included children ages 7.5–11, and measured abilities using a test meant to detect later learning disabilities; thus, they did not utilize an IQ measure. They reported no differences in overall developmental abilities with the exception of auditory discrimination.^16^ Ead et al. only tested 14 children in the United States and found deficits in phonological processing and executive control among children with unilateral hearing loss. While they measured overall IQ, they did not find significant difficulties.^37^ Lastly, Emmett et al., (2014) used NHANES III data of 4823 children ages 6–16 and found decreased nonverbal intelligence scores when comparing typical hearers to age-matched children with bilateral as opposed to unilateral hearing loss, but this group only measured nonverbal IQ using one subtest of an IQ measure, did not report results from the other subtest that they administered, and did not also evaluate verbal IQ.^38^ Two studies of fairly large groups of children (>1000 and >6000, respectively) did find decreased verbal working memory in children with mild hearing loss, but again, did not investigate other abilities.^39^^,^^40^

While we had almost 50% of participants with unilateral hearing impairment, one of the limitations of the study was that we were unable to analyze the effects on unilateral hearing loss on cognition. Were we to stratify our sample by unilateral and bilateral hearing loss, we would be limited to commenting on the results separately, as it is statistically invalid to compare results across stratified models. Alternatively, if we created a new variable with many levels (e.g., moderate/severe hearing loss bilateral, moderate/severe hearing loss unilateral, mild hearing loss bilateral, mild hearing loss unilateral, no hearing loss), the sample sizes in each group would be small, such that multivariate analyses would produce very large confidence intervals (and problems with model stability and convergence). Finally, we group all subjects together, which actually biases the model estimate toward the mean since there are more children with unilateral vs. bilateral hearing loss. Thus, our estimates are conservative, yet we still find an effect in the current analyses. Given that the present study was not designed to examine hypotheses about the differential effects of unilateral and bilateral hearing loss, we believe answering the question of laterality is better suited to a more focused study of subjects with known unilateral and bilateral hearing loss, where cognitive development could be studied in a more controlled manner.

In our study, models that included an interaction term between ear disease and hearing loss also showed no significant interaction effect, suggesting that the effects of ear disease and hearing loss on cognition are additive. Children with both conditions have the lowest estimated cognitive scores in the cohort. Given that such a large proportion of our cohort showed signs of mild (36%) or moderate/severe ear disease (8%) and mild (6%) or moderate/severe hearing loss (2%), the potential burden of cognitive impairment among children in similar settings is enormous. Untreated ear disease/hearing loss is another significant risk factor that is highly likely to interfere with children reaching their learning potential. With no intervention over time, we can still detect effects over a decade later. Thus, the thinking that children will “catch up” due to classroom and/or elective social interaction is flawed. Instead, children with ear disease/hearing loss find themselves on a different trajectory of increased hardship compared to same age peers, likely driven by not only lower language abilities, but reduced reasoning, attention and processing speed, and learning and memory; aspects of cognitive functioning that may not be amenable to intervention. Public health efforts to prevent, diagnose and treat ear disease early would therefore have secondary effects of supporting children's overall cognitive development. By reducing or limiting barriers to the acquisition of oral language, reading skills have a greater chance to develop normally since reading acquisition is based on the phonological or sound system of a child's language. In children with ear disease/hearing loss, since the phonological system is less than adequate, reading skills develop at a slower rate. Without the vocabulary to support decoding of words, hearing impaired readers also struggle with comprehension. When comprehension is compromised readers typically choose to read less. Since hearing impaired readers are likely reading fewer words, their vocabulary and overall background knowledge stagnates. Ear and hearing screenings are not routinely conducted in LMIC, and children with hearing deficits are rarely diagnosed and provided with assistive devices such as hearing aids. Further, children may be provided with interventions only at young ages, or consisting only of speech/language intervention, which is inadequate. Given the significant effects of ear disease and hearing on cognition, our study lends support to one potential mechanism that explains why children with hearing loss are more likely to be unemployed in young adulthood^41^ and about half as likely to obtain higher education.^42^

While the effects of ear disease and hearing loss are notable on their own, the multivariate modeling results also indicate that a variety of family and household characteristics have a significant impact on cognitive development. The univariate results indicate significant differences in family/child and household characteristics between children with no ear disease/hearing loss and moderate to severe ear disease/hearing loss. Given the cross-sectional nature of this study, we cannot conclude if these differences were present in early childhood and therefore contributed to the child's ear and hearing problems. However, the fact that children with ear disease and hearing loss were less likely to attend preschool, more likely to come from crowded and lower SES households, and more likely to live in urban areas, is noteworthy. Parental education and occupation were also higher among children with no ear disease or hearing loss. Children from higher SES families may have received better preventive health care during childhood, thereby decreasing their change of developing ear disease or hearing loss. A comprehensive neuropsychological evaluation typically also includes measures of executive functioning and social-emotional development. Here, we do not report these measures because our response rate for these instruments was lower compared to the response rate of the other cognitive measures collected. Executive functioning (i.e., planning/organization, novel problem-solving, multitasking, task initiation and completion) often plays a significant role in quality of life and is related to both internalizing and externalizing behaviors. Since our study is a longitudinal follow up, the possibility that these multiple intervening factors (i.e., socioeconomic factors) could also independently impact cognition throughout the life course is highly likely as they are known risk factors.

## Contributors

EAS, ASM, ER, VT, and ML designed the original study. ASM devised and supported the cognitive testing. PCL designed the study REDCap databases and coded and classified hearing loss and ear disease per the study protocol. KC devised the classification algorithm for ear disease and supported the hearing testing. VT, ML, and DS ran the field study. PLC, DS, YY, and ER had access to the raw data. PCL and ER verified the data. ER designed and carried out the statistical analysis. YY assisted with statistical analysis. EAS had the final responsibility for the decision to submit for publication. ASM and ER wrote the first draft of the manuscript. All authors reviewed the first draft and contributed revisions to the final manuscript.

## Data sharing statement

Study data that will be made available include the following: Aggregate data that underlie the results reported in this article, after de-identification (text, tables, figures, and appendices); additional, related documents will be available, i.e., study protocol, and statistical analysis plan. These data will be available with publication. Data will be shared and available beginning 9 months and ending 36 months following article publication from the authors, and to researchers who provide a methodologically sound proposal whose proposed use of the data, for metanalysis, has been approved by an independent review committee, and ethical review and clearance by the RITM and AFTMI (Manila). Investigator support will be required after approval of a proposal, with a signed data access agreement.

## Declaration of interests

ASM reports consulting fees from Biogen, Inc., and a grant from the STXBP1 Foundation, which are not related to the current work. EAFS reports grants and consulting fees to the institution from Merck & Co. and Pfizer Inc; grants to the institution from Astra Zeneca Inc., Roche Pharmaceuticals, and Johnson and Johnson; consulting fees to the institution from sanofi paseur, Cidara Therapeutics, Adiago Therapeutics and Nuance Pharmaceuticals; manuscript writing support from Pfizer Inc. and Astra Zeneca Inc.; support for attending a meeting Astra Zeneca Inc. and Pfizer Inc.; and participation on a DSMB from Abbvie Inc, GlaxoSmithKline plc, and Bill and Melinda Gates Foundation, all outside the submitted work. There are no other potential conflicts of interest related to this study.
