# Supplementary material for: Ear disease, hearing loss, and cognitive outcomes in high school children who were previous participants in a randomized placebo controlled trial of an 11 valent conjugate pneumococcal vaccine administered in infancy
Source: Lancet Reg Health West Pac. 2024 Jul 31;49:101128. doi: 10.1016/j.lanwpc.2024.101128 (PMC11459003; doi:10.1016/j.lanwpc.2024.101128)
Supplement: Supplementary Material [file mmc1.docx]

**Supplementary Material**

**Table of Contents**

**Supplementary Table 1**

**Table S1. Univariate regression model results by cognitive domain Page 2**

**Table S1. Univariate regression model results by cognitive domain continued Page 3**

**Supplementary Table 2**

**Table S2. Full multivariate regression model output for each cognitive outcome Page 4**

**Table S2. Full multivariate regression model output for each cognitive outcome continued Page 5**

**Additional References Page 6**

Table S1. Univariate regression model results by cognitive domain

Estimates (in standard deviation units), 95% confidence intervals, and p-values for univariates regression models of full scale IQ, cognitive sub-scales by child and family characteristics, ear disease and hearing loss.

|  | **Full Scale IQ** | | | **VCI** | | | **VSI** | | | **FRI** | | | **WMI** | | | **PSI** | | | **Story learn** | | |
| --- | --- | --- | --- | --- | --- | --- | --- | --- | --- | --- | --- | --- | --- | --- | --- | --- | --- | --- | --- | --- | --- |
|  | **Est** | **SE** | **p-value** | **Est** | **SE** | **p-value** | **Est** | **SE** | **p-value** | **Est** | **SE** | **p-value** | **Est** | **SE** | **p-value** | **Est** | **SE** | **p-value** | **Est** | **SE** | **p-value** |
| Child's birthweight | 0.000 | 0.000 | 0.015 | 0.000 | 0.000 | 0.879 | 0.000 | 0.000 | <.0001 | 0.000 | 0.000 | 0.000 | 0.000 | 0.000 | 0.003 | 0.000 | 0.000 | 0.744 | 0.000 | 0.000 | 0.751 |
| Number of children <15 years in the household | -0.073 | 0.007 | <.0001 | -0.052 | 0.007 | <.0001 | -0.039 | 0.007 | <.0001 | -0.057 | 0.007 | <.0001 | -0.043 | 0.007 | <.0001 | -0.056 | 0.007 | <.0001 | -0.050 | 0.007 | <.0001 |
| Number of adults >15 years in the household | -0.013 | 0.006 | 0.029 | -0.020 | 0.006 | 0.001 | -0.008 | 0.006 | 0.183 | -0.012 | 0.006 | 0.046 | -0.018 | 0.006 | 0.002 | -0.005 | 0.006 | 0.375 | -0.014 | 0.006 | 0.020 |
| Number of people in the household | -0.033 | 0.004 | <.0001 | -0.029 | 0.004 | <.0001 | -0.018 | 0.004 | <.0001 | -0.026 | 0.004 | <.0001 | -0.025 | 0.004 | <.0001 | -0.022 | 0.004 | <.0001 | -0.025 | 0.004 | <.0001 |
| Total births to mother | -0.075 | 0.005 | <.0001 | -0.060 | 0.005 | <.0001 | -0.040 | 0.005 | <.0001 | -0.064 | 0.005 | <.0001 | -0.051 | 0.005 | <.0001 | -0.051 | 0.005 | <.0001 | -0.049 | 0.005 | <.0001 |
| Household SES Index | 0.326 | 0.011 | <.0001 | 0.167 | 0.011 | <.0001 | 0.218 | 0.011 | <.0001 | 0.291 | 0.011 | <.0001 | 0.206 | 0.011 | <.0001 | 0.254 | 0.011 | <.0001 | 0.194 | 0.011 | <.0001 |
| Crowding | -0.100 | 0.007 | <.0001 | -0.069 | 0.007 | <.0001 | -0.062 | 0.006 | <.0001 | -0.085 | 0.006 | <.0001 | -0.068 | 0.006 | <.0001 | -0.084 | 0.006 | <.0001 | -0.072 | 0.006 | <.0001 |
| Child has a chronic health condition | -0.064 | 0.067 | 0.336 | 0.023 | 0.067 | 0.729 | -0.166 | 0.064 | 0.010 | -0.192 | 0.065 | 0.003 | -0.197 | 0.064 | 0.002 | -0.208 | 0.064 | 0.001 | -0.030 | 0.065 | 0.652 |
| Child attended preschool | 0.317 | 0.022 | <.0001 | 0.176 | 0.023 | <.0001 | 0.204 | 0.022 | <.0001 | 0.299 | 0.022 | <.0001 | 0.209 | 0.022 | <.0001 | 0.245 | 0.022 | <.0001 | 0.205 | 0.022 | <.0001 |
| Mother has health insurance | 0.186 | 0.025 | <.0001 | 0.123 | 0.025 | <.0001 | 0.169 | 0.025 | <.0001 | 0.189 | 0.025 | <.0001 | 0.120 | 0.025 | <.0001 | 0.132 | 0.024 | <.0001 | 0.106 | 0.025 | <.0001 |
| Child birth order |  |  |  |  |  |  |  |  |  |  |  |  |  |  |  |  |  |  |  |  |  |
| First | Ref. |  |  | Ref. |  |  | Ref. |  |  | Ref. |  |  | Ref. |  |  | Ref. |  |  | Ref. |  |  |
| Second to fourth | -0.111 | 0.026 | <.0001 | -0.139 | 0.026 | <.0001 | -0.023 | 0.026 | 0.368 | -0.103 | 0.026 | <.0001 | -0.104 | 0.026 | <.0001 | -0.049 | 0.026 | 0.056 | -0.125 | 0.026 | <.0001 |
| Fifth or more | -0.330 | 0.033 | <.0001 | -0.252 | 0.034 | <.0001 | -0.169 | 0.033 | <.0001 | -0.321 | 0.033 | <.0001 | -0.272 | 0.033 | <.0001 | -0.227 | 0.033 | <.0001 | -0.223 | 0.033 | <.0001 |
| Parent's marital status |  |  |  |  |  |  |  |  |  |  |  |  |  |  |  |  |  |  |  |  |  |
| Never married | -0.353 | 0.059 | <.0001 | -0.184 | 0.059 | 0.002 | -0.256 | 0.058 | <.0001 | -0.284 | 0.058 | <.0001 | -0.193 | 0.058 | 0.001 | -0.248 | 0.057 | <.0001 | -0.263 | 0.058 | <.0001 |
| Separated/divorced/widowed | -0.131 | 0.033 | <.0001 | -0.071 | 0.034 | 0.033 | -0.122 | 0.033 | 0.000 | -0.196 | 0.033 | <.0001 | -0.167 | 0.033 | <.0001 | -0.145 | 0.033 | <.0001 | -0.078 | 0.033 | 0.017 |
| Married | Ref. |  |  | Ref. |  |  | Ref. |  |  | Ref. |  |  | Ref. |  |  | Ref. |  |  | Ref. |  |  |
| Parental education highest |  |  |  |  |  |  |  |  |  |  |  |  |  |  |  |  |  |  |  |  |  |
| College or post grad | 0.937 | 0.035 | <.0001 | 0.621 | 0.037 | <.0001 | 0.602 | 0.036 | <.0001 | 0.746 | 0.036 | <.0001 | 0.588 | 0.036 | <.0001 | 0.650 | 0.035 | <.0001 | 0.608 | 0.036 | <.0001 |
| Some college | 0.591 | 0.040 | <.0001 | 0.506 | 0.041 | <.0001 | 0.331 | 0.040 | <.0001 | 0.414 | 0.040 | <.0001 | 0.402 | 0.040 | <.0001 | 0.400 | 0.040 | <.0001 | 0.392 | 0.040 | <.0001 |
| HS or Vocational school grad | 0.413 | 0.034 | <.0001 | 0.367 | 0.036 | <.0001 | 0.235 | 0.035 | <.0001 | 0.260 | 0.035 | <.0001 | 0.289 | 0.035 | <.0001 | 0.285 | 0.034 | <.0001 | 0.285 | 0.035 | <.0001 |
| Elementary school or less | Ref. |  |  | Ref. |  |  | Ref. |  |  | Ref. |  |  | Ref. |  |  | Ref. |  |  | Ref. |  |  |
| Parental employment highest |  |  |  |  |  |  |  |  |  |  |  |  |  |  |  |  |  |  |  |  |  |
| Manager/Professional/Technical | 0.777 | 0.039 | <.0001 | 0.457 | 0.040 | <.0001 | 0.522 | 0.039 | <.0001 | 0.655 | 0.039 | <.0001 | 0.428 | 0.039 | <.0001 | 0.572 | 0.039 | <.0001 | 0.504 | 0.039 | <.0001 |
| Clerical/Service/Armed Forces | 0.272 | 0.032 | <.0001 | 0.230 | 0.033 | <.0001 | 0.162 | 0.032 | <.0001 | 0.204 | 0.032 | <.0001 | 0.131 | 0.032 | <.0001 | 0.224 | 0.032 | <.0001 | 0.214 | 0.032 | <.0001 |
| Agricultural/Crafts/Skilled manual labor/Plant or machine operator or assembler | 0.115 | 0.037 | 0.002 | 0.130 | 0.038 | 0.001 | 0.094 | 0.038 | 0.012 | 0.050 | 0.037 | 0.184 | 0.031 | 0.037 | 0.410 | 0.134 | 0.037 | 0.000 | 0.125 | 0.037 | 0.001 |
| Unskilled manual labor/Farmer | Ref. |  |  | Ref. |  |  | Ref. |  |  | Ref. |  |  | Ref. |  |  | Ref. |  |  |  |  |  |
| Missing | 0.224 | 0.047 | <.0001 | 0.218 | 0.048 | <.0001 | 0.097 | 0.047 | 0.039 | 0.107 | 0.047 | 0.022 | 0.030 | 0.047 | 0.520 | 0.102 | 0.047 | 0.028 | 0.175 | 0.047 | 0.000 |
| Any parent deceased | -0.122 | 0.042 | 0.003 | -0.073 | 0.042 | 0.081 | -0.113 | 0.041 | 0.006 | -0.193 | 0.041 | <.0001 | -0.122 | 0.041 | 0.003 | -0.109 | 0.041 | 0.008 | -0.113 | 0.041 | 0.006 |
| Child lives with |  |  |  |  |  |  |  |  |  |  |  |  |  |  |  |  |  |  |  |  |  |
| One parent | -0.067 | 0.030 | 0.026 | -0.058 | 0.030 | 0.058 | -0.063 | 0.030 | 0.036 | -0.093 | 0.030 | 0.002 | -0.101 | 0.030 | 0.001 | -0.049 | 0.030 | 0.098 | -0.047 | 0.030 | 0.115 |
| Both parents | Ref. |  |  | Ref. |  |  | Ref. |  |  | Ref. |  |  | Ref. |  |  | Ref. |  |  | Ref. |  |  |
| Foster or grandparents, aunt/uncle, sibling, or other | -0.119 | 0.042 | 0.005 | -0.081 | 0.042 | 0.055 | -0.112 | 0.041 | 0.007 | -0.174 | 0.042 | <.0001 | -0.112 | 0.041 | 0.007 | -0.104 | 0.041 | 0.011 | -0.155 | 0.042 | 0.000 |
| Mild Ear Disease | -0.241 | 0.022 | <.0001 | -0.196 | 0.022 | <.0001 | -0.165 | 0.021 | <.0001 | -0.160 | 0.021 | <.0001 | -0.154 | 0.020 | <.0001 | -0.170 | 0.021 | <.0001 | -0.164 | 0.022 | <.0001 |
| Moderate Ear Disease | -0.175 | 0.042 | <.0001 | -0.212 | 0.042 | <.0001 | -0.121 | 0.039 | 0.002 | -0.128 | 0.038 | 0.001 | -0.117 | 0.037 | 0.002 | -0.114 | 0.039 | 0.003 | -0.151 | 0.041 | 0.000 |
| Mild Hearing Loss | -0.372 | 0.041 | <.0001 | -0.347 | 0.042 | <.0001 | -0.243 | 0.039 | <.0001 | -0.318 | 0.037 | <.0001 | -0.298 | 0.037 | <.0001 | -0.299 | 0.038 | <.0001 | -0.229 | 0.041 | <.0001 |
| Moderate Hearing Loss | -0.372 | 0.077 | <.0001 | -0.448 | 0.077 | <.0001 | -0.288 | 0.071 | <.0001 | -0.404 | 0.068 | <.0001 | -0.343 | 0.067 | <.0001 | -0.339 | 0.069 | <.0001 | -0.230 | 0.075 | 0.002 |
| Urban residence | 0.214 | 0.022 | <.0001 | 0.018 | 0.023 | 0.419 | 0.125 | 0.021 | <.0001 | 0.180 | 0.021 | <.0001 | 0.058 | 0.021 | 0.005 | 0.211 | 0.021 | <.0001 | 0.020 | 0.022 | 0.363 |

Note: Ear disease severity was classified accordingly: no ear disease, mild ear disease (acute otitis media, otitis media with effusion, healed perforation of the tympanic membrane, or myringosclerosis), moderate ear disease (dry perforation of the tympanic membrane or adhesive otitis media), or severe ear disease (chronic suppurative otitis media).

Note: Hearing Loss was classified accordingly: no hearing loss, mild hearing loss (16-30 dB PTA), moderate hearing loss (31-60 dB PTA), severe hearing loss (61-80 dB PTA), or profound hearing loss (>80 dB PTA).

Table S1. Univariate regression model results by cognitive domain (continued)

|  | **Story delay** | | | **Verbal list learning** | | | **Verbal list delay** | | | **Picture memory** | | | **Sentence Memory** | | | **Semantic relationships** | | | **Following directions** | | |
| --- | --- | --- | --- | --- | --- | --- | --- | --- | --- | --- | --- | --- | --- | --- | --- | --- | --- | --- | --- | --- | --- |
|  | **Est** | **SE** | **p-value** | **Est** | **SE** | **p-value** | **Est** | **SE** | **p-value** | **Est** | **SE** | **p-value** | **Est** | **SE** | **p-value** | **Est** | **SE** | **p-value** | **Est** | **SE** | **p-value** |
| Child's birthweight | 0.000 | 0.000 | 0.272 | 0.000 | 0.000 | 0.804 | 0.000 | 0.000 | 0.794 | 0.000 | 0.000 | 0.038 | 0.000 | 0.000 | 0.792 | 0.000 | 0.000 | 0.002 | 0.000 | 0.000 | 0.226 |
| Number of children <15 years in the household | -0.055 | 0.007 | <.0001 | -0.004 | 0.007 | 0.594 | -0.001 | 0.007 | 0.852 | -0.018 | 0.007 | 0.010 | -0.018 | 0.007 | 0.013 | -0.055 | 0.007 | <.0001 | -0.049 | 0.007 | <.0001 |
| Number of adults >15 years in the household | -0.016 | 0.006 | 0.008 | 0.009 | 0.006 | 0.146 | 0.005 | 0.006 | 0.384 | 0.007 | 0.006 | 0.233 | -0.012 | 0.006 | 0.038 | -0.011 | 0.006 | 0.063 | -0.016 | 0.006 | 0.005 |
| Number of people in the household | -0.028 | 0.004 | <.0001 | 0.003 | 0.004 | 0.503 | 0.002 | 0.004 | 0.616 | -0.003 | 0.004 | 0.418 | -0.013 | 0.004 | 0.003 | -0.026 | 0.004 | <.0001 | -0.026 | 0.004 | <.0001 |
| Total births to mother | -0.056 | 0.005 | <.0001 | -0.008 | 0.005 | 0.089 | -0.013 | 0.005 | 0.008 | -0.031 | 0.005 | <.0001 | -0.027 | 0.005 | <.0001 | -0.065 | 0.005 | <.0001 | -0.053 | 0.005 | <.0001 |
| Household SES Index | 0.202 | 0.011 | <.0001 | 0.080 | 0.012 | <.0001 | 0.078 | 0.012 | <.0001 | 0.184 | 0.011 | <.0001 | 0.102 | 0.011 | <.0001 | 0.278 | 0.011 | <.0001 | 0.192 | 0.011 | <.0001 |
| Crowding | -0.071 | 0.006 | <.0001 | -0.027 | 0.007 | <.0001 | -0.019 | 0.007 | 0.004 | -0.046 | 0.006 | <.0001 | -0.044 | 0.007 | <.0001 | -0.082 | 0.006 | <.0001 | -0.075 | 0.006 | <.0001 |
| Child has a chronic health condition | -0.042 | 0.065 | 0.516 | -0.105 | 0.066 | 0.112 | -0.005 | 0.066 | 0.941 | 0.039 | 0.064 | 0.547 | -0.071 | 0.066 | 0.277 | -0.018 | 0.064 | 0.774 | -0.217 | 0.065 | 0.001 |
| Child attended preschool | 0.212 | 0.022 | <.0001 | 0.110 | 0.023 | <.0001 | 0.098 | 0.023 | <.0001 | 0.205 | 0.022 | <.0001 | 0.148 | 0.023 | <.0001 | 0.314 | 0.022 | <.0001 | 0.190 | 0.022 | <.0001 |
| Mother has health insurance | 0.091 | 0.025 | 0.000 | 0.060 | 0.025 | 0.017 | 0.050 | 0.025 | 0.048 | 0.108 | 0.024 | <.0001 | 0.064 | 0.025 | 0.010 | 0.156 | 0.024 | <.0001 | 0.151 | 0.025 | <.0001 |
| Child birth order |  |  |  |  |  |  |  |  |  |  |  |  |  |  |  |  |  |  |  |  |  |
| First | Ref. |  |  | Ref. |  |  | Ref. |  |  | Ref. |  |  | Ref. |  |  | Ref. |  |  | Ref. |  |  |
| Second to fourth | -0.126 | 0.026 | <.0001 | -0.034 | 0.026 | 0.194 | -0.036 | 0.026 | 0.168 | -0.071 | 0.025 | 0.005 | -0.095 | 0.026 | 0.000 | -0.103 | 0.025 | <.0001 | -0.084 | 0.026 | 0.001 |
| Fifth or more | -0.258 | 0.033 | <.0001 | -0.041 | 0.034 | 0.226 | -0.088 | 0.034 | 0.009 | -0.169 | 0.033 | <.0001 | -0.156 | 0.033 | <.0001 | -0.267 | 0.033 | <.0001 | -0.252 | 0.033 | <.0001 |
| Parent's marital status |  |  |  |  |  |  |  |  |  |  |  |  |  |  |  |  |  |  |  |  |  |
| Never married | -0.195 | 0.058 | 0.001 | -0.187 | 0.059 | 0.002 | -0.106 | 0.059 | 0.072 | -0.125 | 0.057 | 0.028 | -0.148 | 0.058 | 0.011 | -0.208 | 0.057 | 0.000 | -0.122 | 0.058 | 0.037 |
| Separated/divorced/widowed | -0.060 | 0.033 | 0.068 | -0.081 | 0.034 | 0.015 | -0.063 | 0.034 | 0.059 | 0.012 | 0.032 | 0.703 | -0.056 | 0.033 | 0.090 | -0.092 | 0.032 | 0.005 | -0.144 | 0.033 | <.0001 |
| Married | Ref. |  |  | Ref. |  |  | Ref. |  |  | Ref. |  |  | Ref. |  |  | Ref. |  |  | Ref. |  |  |
| Parental education highest |  |  |  |  |  |  |  |  |  |  |  |  |  |  |  |  |  |  |  |  |  |
| College or post grad | 0.638 | 0.036 | <.0001 | 0.302 | 0.037 | <.0001 | 0.321 | 0.037 | <.0001 | 0.590 | 0.035 | <.0001 | 0.402 | 0.037 | <.0001 | 0.695 | 0.035 | <.0001 | 0.560 | 0.036 | <.0001 |
| Some college | 0.417 | 0.040 | <.0001 | 0.270 | 0.042 | <.0001 | 0.269 | 0.042 | <.0001 | 0.358 | 0.040 | <.0001 | 0.327 | 0.041 | <.0001 | 0.348 | 0.039 | <.0001 | 0.345 | 0.041 | <.0001 |
| HS or Vocational school grad | 0.297 | 0.035 | <.0001 | 0.216 | 0.036 | <.0001 | 0.189 | 0.036 | <.0001 | 0.274 | 0.034 | <.0001 | 0.259 | 0.036 | <.0001 | 0.208 | 0.034 | <.0001 | 0.306 | 0.035 | <.0001 |
| Elementary school or less | Ref. |  |  | Ref. |  |  | Ref. |  |  | Ref. |  |  | Ref. |  |  | Ref. |  |  | Ref. |  |  |
| Parental employment highest |  |  |  |  |  |  |  |  |  |  |  |  |  |  |  |  |  |  |  |  |  |
| Manager/Professional/Technical | 0.505 | 0.039 | <.0001 | 0.210 | 0.040 | <.0001 | 0.204 | 0.040 | <.0001 | 0.447 | 0.038 | <.0001 | 0.249 | 0.040 | <.0001 | 0.639 | 0.038 | <.0001 | 0.436 | 0.039 | <.0001 |
| Clerical/Service/Armed Forces | 0.208 | 0.032 | <.0001 | 0.143 | 0.033 | <.0001 | 0.122 | 0.033 | 0.000 | 0.155 | 0.032 | <.0001 | 0.074 | 0.033 | 0.025 | 0.167 | 0.032 | <.0001 | 0.169 | 0.033 | <.0001 |
| Agricultural/Crafts/Skilled manual labor/Plant or machine operator or assembler | 0.110 | 0.037 | 0.003 | 0.088 | 0.038 | 0.021 | 0.079 | 0.038 | 0.040 | 0.075 | 0.037 | 0.042 | 0.033 | 0.038 | 0.388 | 0.048 | 0.037 | 0.195 | 0.098 | 0.038 | 0.010 |
| Unskilled manual labor/Farmer |  |  |  |  |  |  |  |  |  |  |  |  |  |  |  | Ref. |  |  | Ref. |  |  |
| Missing | 0.209 | 0.047 | <.0001 | 0.139 | 0.049 | 0.004 | 0.105 | 0.049 | 0.031 | 0.097 | 0.047 | 0.038 | 0.033 | 0.048 | 0.488 | 0.225 | 0.046 | <.0001 | 0.103 | 0.047 | 0.029 |
| Any parent deceased | -0.108 | 0.041 | 0.009 | -0.075 | 0.042 | 0.073 | -0.048 | 0.042 | 0.254 | -0.050 | 0.040 | 0.213 | -0.096 | 0.041 | 0.021 | -0.124 | 0.041 | 0.002 | -0.124 | 0.041 | 0.003 |
| Child lives with |  |  |  |  |  |  |  |  |  |  |  |  |  |  |  |  |  |  |  |  |  |
| One parent | -0.022 | 0.030 | 0.456 | -0.041 | 0.030 | 0.172 | -0.034 | 0.030 | 0.260 | 0.037 | 0.029 | 0.206 | -0.055 | 0.030 | 0.065 | -0.038 | 0.030 | 0.203 | -0.059 | 0.030 | 0.048 |
| Both parents | Ref. |  |  | Ref. |  |  | Ref. |  |  | Ref. |  |  | Ref. |  |  | Ref. |  |  | Ref. |  |  |
| Foster or grandparents, aunt/uncle, sibling, or other | -0.139 | 0.041 | 0.001 | -0.140 | 0.042 | 0.001 | -0.074 | 0.042 | 0.080 | -0.076 | 0.041 | 0.064 | -0.041 | 0.042 | 0.324 | -0.118 | 0.041 | 0.004 | -0.099 | 0.041 | 0.018 |
| Mild Ear Disease | -0.157 | 0.022 | <.0001 | -0.098 | 0.022 | <.0001 | -0.073 | 0.022 | 0.001 | -0.079 | 0.022 | 0.000 | -0.146 | 0.022 | <.0001 | -0.191 | 0.022 | <.0001 | -0.153 | 0.021 | <.0001 |
| Moderate Ear Disease | -0.153 | 0.041 | 0.000 | -0.079 | 0.042 | 0.058 | -0.046 | 0.042 | 0.274 | -0.091 | 0.040 | 0.024 | -0.211 | 0.041 | <.0001 | -0.173 | 0.041 | <.0001 | -0.174 | 0.038 | <.0001 |
| Mild Hearing Loss | -0.255 | 0.041 | <.0001 | -0.258 | 0.041 | <.0001 | -0.283 | 0.041 | <.0001 | -0.162 | 0.040 | <.0001 | -0.221 | 0.041 | <.0001 | -0.310 | 0.040 | <.0001 | -0.303 | 0.038 | <.0001 |
| Moderate Hearing Loss | -0.239 | 0.075 | 0.001 | -0.196 | 0.076 | 0.010 | -0.214 | 0.076 | 0.005 | -0.094 | 0.073 | 0.200 | -0.273 | 0.076 | 0.000 | -0.343 | 0.074 | <.0001 | -0.409 | 0.069 | <.0001 |
| Urban residence | 0.056 | 0.022 | 0.012 | -0.053 | 0.023 | 0.020 | -0.023 | 0.023 | 0.310 | 0.199 | 0.022 | <.0001 | -0.013 | 0.023 | 0.564 | 0.207 | 0.022 | <.0001 | 0.039 | 0.021 | 0.064 |

Note: Ear disease severity was classified accordingly: no ear disease, mild ear disease (acute otitis media, otitis media with effusion, healed perforation of the tympanic membrane, or myringosclerosis), moderate ear disease (dry perforation of the tympanic membrane or adhesive otitis media), or severe ear disease (chronic suppurative otitis media).

Note: Hearing Loss was classified accordingly: no hearing loss, mild hearing loss (16-30 dB PTA), moderate hearing loss (31-60 dB PTA), severe hearing loss (61-80 dB PTA), or profound hearing loss (>80 dB PTA).

Table S2. Full multivariate regression model output for each cognitive outcome

Estimates (in standard deviation units), 95% confidence intervals, and p-values for multivariate regression models of full scale IQ and cognitive sub-scales. Models are adjusted for all covariates listed in the first column including mild and moderate/severe ear disease (also reported in Figure 2 in the main paper) and middle and moderate/severe/profound hearing loss (also reported in Figure 3 in the main paper).

|  | ***Model*** | | | | | | | | | | | | | |
| --- | --- | --- | --- | --- | --- | --- | --- | --- | --- | --- | --- | --- | --- | --- |
|  | **Full IQ** | **VCI** | **VSI** | **FRI** | **WMI** | **PSI** | **Story learn** | **Story delay** | **Verbal list learning** | **Verbal list delay** | **Picture memory** | **Sentence memory** | **Semantic reasoning** | **Following directions** |
| **Observations (n)** | 7,875 | 7,942 | 7,970 | 7,967 | 7,964 | 7,971 | 8,007 | 8,004 | 8,010 | 7,994 | 8,009 | 8,008 | 7,967 | 7,966 |
| **Variable** |  |  |  |  |  |  |  |  |  |  |  |  |  |  |
| **Mild ear disease** |  |  |  |  |  |  |  |  |  |  |  |  |  |  |
| Estimate | -0.152 | -0.121 | -0.115 | -0.081 | -0.095 | -0.106 | -0.094 | -0.082 | -0.071 | -0.05 | -0.031 | -0.09 | -0.106 | -0.083 |
| 95% CI | -0.196, -0.108 | -0.168, -0.075 | -0.158, -0.072 | -0.122, -0.040 | -0.136, -0.053 | -0.148, -0.064 | -0.139, -0.048 | -0.127, -0.037 | -0.118, -0.024 | -0.097, -0.002 | -0.076, 0.014 | -0.137, -0.043 | -0.151, -0.062 | -0.126, -0.041 |
| p-value | 0.000 | 0.00000 | 0.00000 | 0.0002 | 0.00001 | 0.00000 | 0.0001 | 0.0004 | 0.004 | 0.040 | 0.174 | 0.0002 | 0.00001 | 0.0002 |
| **Moderate/severe ear disease** |  |  |  |  |  |  |  |  |  |  |  |  |  |  |
| Estimate | -0.11 | -0.145 | -0.095 | -0.046 | -0.045 | -0.056 | -0.1 | -0.086 | -0.037 | 0.015 | -0.064 | -0.179 | -0.116 | -0.104 |
| 95% CI | -0.191, -0.029 | -0.231, -0.060 | -0.174, -0.015 | -0.121, 0.029 | -0.121, 0.031 | -0.133, 0.021 | -0.184, -0.017 | -0.169, -0.003 | -0.124, 0.050 | -0.072, 0.102 | -0.147, 0.018 | -0.265, -0.093 | -0.197, -0.035 | -0.182, -0.026 |
| p-value | 0.008 | 0.001 | 0.020 | 0.230 | 0.248 | 0.155 | 0.019 | 0.043 | 0.410 | 0.735 | 0.127 | 0.00005 | 0.006 | 0.010 |
| **Mild hearing loss** |  |  |  |  |  |  |  |  |  |  |  |  |  |  |
| Estimate | -0.233 | -0.22 | -0.134 | -0.169 | -0.2 | -0.183 | -0.143 | -0.174 | -0.246 | -0.281 | -0.1 | -0.134 | -0.167 | -0.18 |
| 95% CI | -0.322, -0.143 | -0.314, -0.126 | -0.221, -0.046 | -0.252, -0.087 | -0.284, -0.117 | -0.268, -0.098 | -0.235, -0.051 | -0.266, -0.082 | -0.342, -0.150 | -0.377, -0.185 | -0.191, -0.009 | -0.228, -0.039 | -0.257, -0.078 | -0.266, -0.094 |
| p-value | 0.00000 | 0.00001 | 0.003 | 0.0001 | 0.00001 | 0.00003 | 0.003 | 0.0002 | 0.00000 | 0.000 | 0.032 | 0.006 | 0.0003 | 0.00004 |
| **Moderate/severe/profound hearing loss** | |  |  |  |  |  |  |  |  |  |  |  |  |  |
| Estimate | -0.276 | -0.387 | -0.219 | -0.327 | -0.293 | -0.264 | -0.167 | -0.174 | -0.181 | -0.211 | -0.025 | -0.205 | -0.251 | -0.353 |
| 95% CI | -0.418, -0.134 | -0.536, -0.238 | -0.355, -0.083 | -0.456, -0.199 | -0.423, -0.163 | -0.396, -0.132 | -0.313, -0.022 | -0.318, -0.030 | -0.332, -0.031 | -0.361, -0.060 | -0.168, 0.118 | -0.354, -0.057 | -0.390, -0.111 | -0.487, -0.219 |
| p-value | 0.0002 | 0.00000 | 0.002 | 0.00000 | 0.00001 | 0.0001 | 0.024 | 0.019 | 0.019 | 0.007 | 0.730 | 0.007 | 0.0005 | 0.00000 |
| **Household SES index** |  |  |  |  |  |  |  |  |  |  |  |  |  |  |
| Estimate | 0.171 | 0.064 | 0.107 | 0.145 | 0.106 | 0.135 | 0.089 | 0.097 | 0.045 | 0.039 | 0.099 | 0.032 | 0.133 | 0.09 |
| 95% CI | 0.142, 0.201 | 0.033, 0.095 | 0.078, 0.137 | 0.118, 0.173 | 0.078, 0.134 | 0.107, 0.164 | 0.059, 0.120 | 0.067, 0.127 | 0.013, 0.077 | 0.007, 0.071 | 0.068, 0.129 | 0.0004, 0.063 | 0.103, 0.163 | 0.061, 0.119 |
| p-value | 0.000 | 0.0001 | 0.000 | 0.000 | 0.000 | 0.000 | 0.000 | 0.000 | 0.006 | 0.017 | 0.000 | 0.047 | 0.000 | 0.000 |
| **Household crowding** |  |  |  |  |  |  |  |  |  |  |  |  |  |  |
| Estimate | -0.016 | -0.013 | -0.008 | -0.013 | -0.014 | -0.029 | -0.021 | -0.014 | -0.007 | 0.003 | -0.003 | -0.018 | -0.01 | -0.024 |
| 95% CI | -0.031, -0.002 | -0.028, 0.002 | -0.022, 0.006 | -0.027, -0.0001 | -0.027, -0.0004 | -0.043, -0.015 | -0.035, -0.006 | -0.029, 0.0005 | -0.022, 0.008 | -0.013, 0.018 | -0.018, 0.011 | -0.033, -0.003 | -0.024, 0.004 | -0.038, -0.011 |
| p-value | 0.026 | 0.096 | 0.269 | 0.049 | 0.043 | 0.00004 | 0.007 | 0.059 | 0.378 | 0.736 | 0.676 | 0.021 | 0.172 | 0.001 |
| **Total births to mother** |  |  |  |  |  |  |  |  |  |  |  |  |  |  |
| Estimate | -0.02 | -0.037 | -0.004 | -0.013 | -0.013 | -0.003 | -0.013 | -0.021 | 0.008 | 0.007 | 0.006 | 0.001 | -0.027 | -0.018 |
| 95% CI | -0.034, -0.007 | -0.052, -0.023 | -0.017, 0.010 | -0.026, -0.0003 | -0.026, -0.00005 | -0.016, 0.010 | -0.028, 0.001 | -0.035, -0.007 | -0.006, 0.023 | -0.008, 0.021 | -0.008, 0.021 | -0.014, 0.015 | -0.041, -0.013 | -0.032, -0.005 |
| p-value | 0.004 | 0.00000 | 0.606 | 0.045 | 0.050 | 0.690 | 0.063 | 0.004 | 0.266 | 0.374 | 0.365 | 0.915 | 0.0002 | 0.007 |
| **Child attended preschool** |  |  |  |  |  |  |  |  |  |  |  |  |  |  |
| Estimate | 0.017 | 0.017 | -0.006 | 0.025 | 0.015 | 0.008 | 0.026 | 0.021 | 0.051 | 0.029 | 0.024 | 0.061 | 0.06 | 0.012 |
| 95% CI | -0.028, 0.063 | -0.031, 0.065 | -0.051, 0.039 | -0.017, 0.068 | -0.028, 0.058 | -0.036, 0.052 | -0.021, 0.073 | -0.026, 0.068 | 0.002, 0.100 | -0.020, 0.078 | -0.022, 0.071 | 0.012, 0.109 | 0.014, 0.106 | -0.033, 0.056 |
| p-value | 0.454 | 0.481 | 0.781 | 0.243 | 0.491 | 0.716 | 0.281 | 0.383 | 0.042 | 0.251 | 0.308 | 0.014 | 0.011 | 0.605 |
| **Child birth order: second to fourth** | |  |  |  |  |  |  |  |  |  |  |  |  |  |
| Estimate | -0.035 | -0.051 | 0.024 | -0.038 | -0.045 | -0.002 | -0.081 | -0.072 | -0.016 | -0.018 | -0.051 | -0.064 | -0.028 | -0.015 |
| 95% CI | -0.085, 0.016 | -0.105, 0.002 | -0.026, 0.074 | -0.085, 0.009 | -0.092, 0.003 | -0.051, 0.046 | -0.133, -0.028 | -0.125, -0.020 | -0.071, 0.038 | -0.073, 0.037 | -0.103, 0.001 | -0.118, -0.010 | -0.079, 0.024 | -0.064, 0.034 |
| p-value | 0.179 | 0.061 | 0.348 | 0.115 | 0.067 | 0.924 | 0.003 | 0.007 | 0.558 | 0.517 | 0.057 | 0.020 | 0.290 | 0.540 |
| **Child birth order: fifth or more** | |  |  |  |  |  |  |  |  |  |  |  |  |  |
| Estimate | -0.021 | 0.059 | 0.021 | -0.059 | -0.045 | -0.031 | -0.023 | -0.026 | 0.004 | -0.036 | -0.078 | -0.056 | 0.023 | -0.003 |
| 95% CI | -0.107, 0.066 | -0.033, 0.150 | -0.064, 0.107 | -0.139, 0.022 | -0.127, 0.036 | -0.114, 0.052 | -0.113, 0.067 | -0.115, 0.064 | -0.089, 0.098 | -0.130, 0.058 | -0.167, 0.011 | -0.148, 0.037 | -0.064, 0.111 | -0.087, 0.081 |
| p-value | 0.643 | 0.209 | 0.628 | 0.154 | 0.276 | 0.470 | 0.620 | 0.570 | 0.930 | 0.451 | 0.087 | 0.239 | 0.604 | 0.953 |
| **Parental marital status: never married** | |  |  |  |  |  |  |  |  |  |  |  |  |  |
| Estimate | -0.281 | -0.188 | -0.186 | -0.229 | -0.164 | -0.159 | -0.225 | -0.172 | -0.152 | -0.069 | -0.048 | -0.132 | -0.166 | -0.091 |
| 95% CI | -0.391, -0.170 | -0.305, -0.072 | -0.295, -0.078 | -0.331, -0.127 | -0.268, -0.061 | -0.265, -0.054 | -0.338, -0.111 | -0.285, -0.058 | -0.270, -0.033 | -0.188, 0.049 | -0.161, 0.065 | -0.249, -0.016 | -0.277, -0.055 | -0.198, 0.015 |
| p-value | 0.00000 | 0.002 | 0.001 | 0.00002 | 0.002 | 0.003 | 0.0002 | 0.004 | 0.013 | 0.252 | 0.404 | 0.027 | 0.004 | 0.092 |
| **Parental marital status: separated/widowed** | |  |  |  |  |  |  |  |  |  |  |  |  |  |
| Estimate | -0.078 | -0.053 | -0.022 | -0.083 | -0.053 | -0.036 | -0.047 | -0.036 | -0.063 | -0.04 | 0.053 | -0.029 | -0.05 | -0.042 |
| 95% CI | -0.141, -0.014 | -0.120, 0.013 | -0.085, 0.040 | -0.142, -0.024 | -0.113, 0.006 | -0.097, 0.025 | -0.113, 0.018 | -0.101, 0.029 | -0.131, 0.005 | -0.108, 0.028 | -0.011, 0.118 | -0.096, 0.038 | -0.114, 0.014 | -0.103, 0.019 |
| p-value | 0.017 | 0.116 | 0.483 | 0.006 | 0.081 | 0.245 | 0.156 | 0.284 | 0.070 | 0.246 | 0.107 | 0.396 | 0.125 | 0.180 |

Table S2. Full multivariate regression model output for each cognitive outcome (continued)

|  | ***Model*** | | | | | | | | | | | | | |
| --- | --- | --- | --- | --- | --- | --- | --- | --- | --- | --- | --- | --- | --- | --- |
|  | **Full IQ** | **VCI** | **VSI** | **FRI** | **WMI** | **PSI** | **Story learn** | **Story delay** | **Verbal list learning** | **Verbal list delay** | **Picture memory** | **Sentence memory** | **Semantic reasoning** | **Following directions** |
| **Variable** |  |  |  |  |  |  |  |  |  |  |  |  |  |  |
| **Parental education: high school/vocational school graduate** | | |  |  |  |  |  |  |  |  |  |  |  |  |
| Estimate | 0.236 | 0.201 | 0.165 | 0.137 | 0.162 | 0.168 | 0.203 | 0.187 | 0.137 | 0.141 | 0.15 | 0.187 | 0.103 | 0.174 |
| 95% CI | 0.177, 0.295 | 0.139, 0.263 | 0.107, 0.223 | 0.083, 0.192 | 0.107, 0.217 | 0.112, 0.225 | 0.142, 0.263 | 0.126, 0.247 | 0.074, 0.201 | 0.078, 0.204 | 0.090, 0.210 | 0.124, 0.249 | 0.044, 0.163 | 0.117, 0.231 |
| p-value | 0.000 | 0.000 | 0.00000 | 0.00000 | 0.000 | 0.000 | 0.000 | 0.000 | 0.00003 | 0.00002 | 0.00001 | 0.000 | 0.001 | 0.000 |
| **Parental education: some college** | |  |  |  |  |  |  |  |  |  |  |  |  |  |
| Estimate | 0.304 | 0.285 | 0.177 | 0.189 | 0.201 | 0.18 | 0.235 | 0.236 | 0.167 | 0.192 | 0.167 | 0.208 | 0.162 | 0.138 |
| 95% CI | 0.236, 0.371 | 0.215, 0.356 | 0.110, 0.243 | 0.126, 0.251 | 0.137, 0.264 | 0.116, 0.245 | 0.165, 0.305 | 0.167, 0.305 | 0.094, 0.239 | 0.120, 0.265 | 0.098, 0.236 | 0.137, 0.280 | 0.094, 0.230 | 0.073, 0.203 |
| p-value | 0.000 | 0.000 | 0.00000 | 0.000 | 0.000 | 0.00000 | 0.000 | 0.000 | 0.00001 | 0.00000 | 0.00001 | 0.000 | 0.00001 | 0.00004 |
| **Parental education: college graduate** | |  |  |  |  |  |  |  |  |  |  |  |  |  |
| Estimate | 0.388 | 0.276 | 0.294 | 0.308 | 0.243 | 0.238 | 0.31 | 0.311 | 0.139 | 0.201 | 0.266 | 0.195 | 0.281 | 0.221 |
| 95% CI | 0.318, 0.457 | 0.203, 0.349 | 0.225, 0.363 | 0.243, 0.372 | 0.178, 0.309 | 0.172, 0.305 | 0.238, 0.382 | 0.239, 0.383 | 0.064, 0.214 | 0.126, 0.276 | 0.195, 0.338 | 0.121, 0.269 | 0.210, 0.351 | 0.153, 0.288 |
| p-value | 0.000 | 0.000 | 0.000 | 0.000 | 0.000 | 0.000 | 0.000 | 0.000 | 0.0003 | 0.00000 | 0.000 | 0.00000 | 0.000 | 0.000 |
| **Parental employment: Clerical/Service/Armed Forces** | | |  |  |  |  |  |  |  |  |  |  |  |  |
| Estimate | -0.115 | -0.033 | -0.099 | -0.102 | -0.053 | -0.051 | -0.049 | -0.041 | 0.028 | 0.023 | -0.055 | -0.058 | -0.135 | -0.04 |
| 95% CI | -0.184, -0.046 | -0.106, 0.040 | -0.168, -0.031 | -0.166, -0.037 | -0.118, 0.012 | -0.118, 0.015 | -0.121, 0.023 | -0.112, 0.031 | -0.047, 0.102 | -0.052, 0.097 | -0.126, 0.016 | -0.131, 0.016 | -0.205, -0.066 | -0.107, 0.027 |
| p-value | 0.002 | 0.374 | 0.005 | 0.002 | 0.111 | 0.130 | 0.181 | 0.263 | 0.464 | 0.551 | 0.127 | 0.124 | 0.0002 | 0.238 |
| **Parental employment: Agricultural/Crafts/Skilled manual labor/Plant or machine operator or assembler** | | | | |  |  |  |  |  |  |  |  |  |  |
| Estimate | -0.148 | -0.051 | -0.105 | -0.172 | -0.088 | -0.059 | -0.05 | -0.042 | 0.008 | 0.024 | -0.059 | -0.046 | -0.155 | -0.051 |
| 95% CI | -0.229, -0.066 | -0.137, 0.035 | -0.186, -0.024 | -0.248, -0.096 | -0.165, -0.011 | -0.137, 0.020 | -0.135, 0.034 | -0.127, 0.042 | -0.080, 0.097 | -0.064, 0.112 | -0.143, 0.025 | -0.133, 0.041 | -0.237, -0.072 | -0.130, 0.028 |
| p-value | 0.0004 | 0.250 | 0.011 | 0.00001 | 0.026 | 0.142 | 0.244 | 0.326 | 0.851 | 0.591 | 0.170 | 0.300 | 0.0003 | 0.206 |
| **Parental employment: Unskilled manual labor/Farmer** | | |  |  |  |  |  |  |  |  |  |  |  |  |
| Estimate | -0.187 | -0.134 | -0.14 | -0.144 | -0.053 | -0.123 | -0.131 | -0.111 | -0.054 | -0.031 | -0.104 | -0.045 | -0.15 | -0.091 |
| 95% CI | -0.274, -0.101 | -0.226, -0.043 | -0.226, -0.055 | -0.225, -0.064 | -0.135, 0.028 | -0.206, -0.040 | -0.221, -0.041 | -0.200, -0.022 | -0.147, 0.040 | -0.124, 0.063 | -0.193, -0.015 | -0.137, 0.047 | -0.237, -0.063 | -0.175, -0.008 |
| p-value | 0.00003 | 0.004 | 0.002 | 0.0005 | 0.199 | 0.004 | 0.005 | 0.015 | 0.259 | 0.520 | 0.022 | 0.339 | 0.001 | 0.033 |
| **Parental employment: Missing** | |  |  |  |  |  |  |  |  |  |  |  |  |  |
| Estimate | -0.074 | 0.024 | -0.057 | -0.047 | -0.021 | -0.069 | -0.008 | 0.041 | 0.073 | 0.045 | -0.085 | -0.05 | 0.002 | 0.015 |
| 95% CI | -0.171, 0.024 | -0.079, 0.127 | -0.154, 0.039 | -0.139, 0.044 | -0.113, 0.071 | -0.163, 0.025 | -0.110, 0.093 | -0.060, 0.142 | -0.033, 0.179 | -0.061, 0.151 | -0.185, 0.016 | -0.155, 0.054 | -0.096, 0.101 | -0.080, 0.109 |
| p-value | 0.140 | 0.652 | 0.245 | 0.307 | 0.659 | 0.149 | 0.871 | 0.428 | 0.175 | 0.405 | 0.099 | 0.343 | 0.961 | 0.763 |
| **Child lives in urban setting** |  |  |  |  |  |  |  |  |  |  |  |  |  |  |
| Estimate | 0.077 | -0.053 | 0.033 | 0.061 | -0.027 | 0.12 | -0.064 | -0.035 | -0.092 | -0.079 | 0.117 | -0.059 | 0.08 | -0.03 |
| 95% CI | 0.034, 0.120 | -0.098, -0.007 | -0.009, 0.076 | 0.021, 0.101 | -0.068, 0.013 | 0.078, 0.161 | -0.109, -0.020 | -0.079, 0.010 | -0.138, -0.045 | -0.125, -0.032 | 0.073, 0.162 | -0.105, -0.014 | 0.037, 0.124 | -0.071, 0.012 |
| p-value | 0.0005 | 0.023 | 0.125 | 0.004 | 0.191 | 0.000 | 0.005 | 0.128 | 0.0002 | 0.001 | 0.00000 | 0.012 | 0.0003 | 0.164 |
| **Constant** |  |  |  |  |  |  |  |  |  |  |  |  |  |  |
| Estimate | 0.074 | 0.169 | 0.033 | 0.128 | 0.132 | 0.037 | 0.087 | 0.07 | -0.059 | -0.104 | -0.111 | 0.052 | 0.133 | 0.174 |
| 95% CI | -0.026, 0.174 | 0.064, 0.275 | -0.066, 0.132 | 0.035, 0.222 | 0.037, 0.226 | -0.060, 0.133 | -0.017, 0.191 | -0.033, 0.174 | -0.168, 0.049 | -0.212, 0.004 | -0.214, -0.008 | -0.055, 0.159 | 0.032, 0.234 | 0.077, 0.271 |
| p-value | 0.150 | 0.002 | 0.515 | 0.008 | 0.007 | 0.456 | 0.103 | 0.182 | 0.283 | 0.060 | 0.034 | 0.341 | 0.011 | 0.0005 |

Note: Ear disease severity was classified accordingly: no ear disease, mild ear disease (acute otitis media, otitis media with effusion, healed perforation of the tympanic membrane, or myringosclerosis), moderate ear disease (dry perforation of the tympanic membrane or adhesive otitis media), or severe ear disease (chronic suppurative otitis media).

Note: Hearing Loss was classified accordingly: no hearing loss, mild hearing loss (16-30 dB PTA), moderate hearing loss (31-60 dB PTA), severe hearing loss (61-80 dB PTA), or profound hearing loss (>80 dB PTA).

Additional References:

(1-3)

1. Wechsler D. WISC-V Technical and Interpretive Manual. Bloomington, MN: Pearson; 2014.

2. Sheslow DA, W. Wide Range Assessment of Memory and Learning - Second Edition (WRAML-2) Manual. Bloomington, MN: Pearson; 2003.

3. Wiig EH, Semel, E., Secord, W. A. Clinical Evaluation of Language Fundamentals–Fifth Edition (CELF-5). Bloomington, MN: NCS Pearson; 2013.
